# A novel protein AXIN1-295aa encoded by circAXIN1 activates the Wnt/β-catenin signaling pathway to promote gastric cancer progression

**DOI:** 10.1186/s12943-021-01457-w

**Published:** 2021-12-04

**Authors:** Yin Peng, Yidan Xu, Xiaojing Zhang, Shiqi Deng, Yuan Yuan, Xiaonuan Luo, Md Tofazzal Hossain, Xiaohui Zhu, Kaining Du, Fan Hu, Yang Chen, Shanshan Chang, Xianling Feng, Xinmin Fan, Hassan Ashktorab, Duane Smoot, Stephen J. Meltzer, Gangqiang Hou, Yanjie Wei, Song Li, Ying Qin, Zhe Jin

**Affiliations:** 1 Guangdong Provincial Key Laboratory for Genome Stability & Disease Prevention and Regional Immunity and Diseases, Department of Pathology, Shenzhen University School of Medicine, 3688 Nanhai Avenue, Nanshan, Shenzhen, Guangdong 518060 People’s Republic of China; 2 University of Chinese Academy of Sciences, No.19(A) Yuquan Road, Shijingshan District Beijing, 100049 People’s Republic of China; 3 Center for High Performance Computing, Shenzhen Institutes of Advanced Technology, Chinese Academy of Sciences, Shenzhen, Guangdong 518000 People’s Republic of China; 4 Department of Statistics, Bangabandhu Sheikh Mujibur Rahaman Science and Technology University, Gopalganj, 8100 Bangladesh; 5 Department of Medicine and Cancer Center, Howard University, College of Medicine, Washington, DC, 20060 USA; 6 Department of Medicine, Meharry Medical Center, Nashville, TN 37208 USA; 7 Department of Medicine/GI Division, Johns Hopkins University School of Medicine and Sidney Kimmel Comprehensive Cancer Center, Baltimore, MD 21287 USA; 8 Department of Medical Image Center, Kangning Hospital, Shenzhen, Guangdong 518000 People’s Republic of China; 9 Shenzhen Science & Technology Development Exchange Center, Shenzhen Science and Technology Building, Shenzhen, Guangdong 518055 People’s Republic of China; 10 Department of Gastrointestinal Surgery, Shenzhen Second People’s Hospital, Shenzhen, Guangdong 518000 People’s Republic of China

**Keywords:** AXIN1, Wnt, Translation, circRNA

## Abstract

**Background:**

Circular RNA (circRNA), a subclass of non-coding RNA, plays a critical role in cancer tumorigenesis and metastasis. It has been suggested that circRNA acts as a microRNA sponge or a scaffold to interact with protein complexes; however, its full range of functions remains elusive. Recently, some circRNAs have been found to have coding potential.

**Methods:**

To investigate the role of circRNAs in gastric cancer (GC), parallel sequencing was performed using five paired GC samples. Differentially expressed circAXIN1 was proposed to encode a novel protein. FLAG-tagged circRNA overexpression plasmid construction, immunoblotting, mass spectrometry, and luciferase reporter analyses were applied to confirm the coding potential of circAXIN1. Gain- and loss-of-function studies were conducted to study the oncogenic role of circAXIN1 and AXIN1-295aa on the proliferation, migration, invasion, and metastasis of GC cells in vitro and in vivo. The competitive interaction between AXIN1-295aa and adenomatous polyposis coli (APC) was investigated by immunoprecipitation analyses. Wnt signaling activity was observed using a Top/Fopflash assay, real-time quantitative RT-PCR, immunoblotting, immunofluorescence staining, and chromatin immunoprecipitation.

**Results:**

CircAXIN1 is highly expressed in GC tissues compared with its expression in paired adjacent normal gastric tissues. CircAXIN1 encodes a 295 amino acid (aa) novel protein, which was named AXIN1-295aa. CircAXIN1 overexpression enhances the cell proliferation, migration, and invasion of GC cells, while the knockdown of circAXIN1 inhibits the malignant behaviors of GC cells in vitro and in vivo*.* Mechanistically, AXIN1-295aa competitively interacts with APC, leading to dysfunction of the “destruction complex” of the Wnt pathway. Released β-catenin translocates to the nucleus and binds to the TCF consensus site on the promoter, inducing downstream gene expression.

**Conclusion:**

CircAXIN1 encodes a novel protein, AXIN1-295aa. AXIN1-295aa functions as an oncogenic protein, activating the Wnt signaling pathway to promote GC tumorigenesis and progression, suggesting a potential therapeutic target for GC.

**Supplementary Information:**

The online version contains supplementary material available at 10.1186/s12943-021-01457-w.

## Background

Circular RNAs (circRNAs) are a class of transcripts characterized by a covalently closed loop structure. They have no 5′ to 3′ polarity or polyA tail [1]. Most circRNAs are produced by the back splicing of exons, a non-canonical splicing process [2, 3]. CircRNAs are expressed in a tissue-specific, developmental stage-specific, and disease-specific manner [4]. They are known to act as microRNA sponges, transcription regulators, and scaffolds for mediating protein interactions or localization [5]. More recently, circRNAs were found to harbor coding potential [6–8], which is reasonable, as most circRNAs contain exons and are localized in the cytoplasm [9]. Zhang’s group has previously reported that a panel of circRNAs are translated into proteins that function as tumor suppressors in glioblastomas [10].

Gastric cancer (GC) has a high mortality rate in China and around the world due to the lack of efficient tools for early diagnosis [11]. The high stability of circRNA makes it a good candidate as a molecular biomarker for early diagnosis [1]. The Wnt/β-catenin signaling pathway plays an important role in normal embryo development, tissue differentiation, homeostasis, and oncogenesis [12]. Mutations in Wnt signaling are observed in the majority of cancers, as it is essential for the viability of cells [13]. Changes in the stability of cytoplasmic β-catenin is a key switch in the Wnt pathway [13], which is monitored by the APC/AXIN destruction complex [14]. The dysfunction of APC [15] or AXIN [16] can lead to abnormal β-catenin accumulation. Furthermore, the Wnt/β-catenin signaling pathway is aberrantly active in 30 to 50% of patients with GC [17, 18], although the mechanism underlying abnormal β-catenin activation in GC is unclear. There has been little research that has addressed the functional role of circRNA in GC development [19, 20]. Exploring the function and regulatory role of circRNA in the Wnt/β-catenin signaling pathway will facilitate a better understanding of the molecular pathogenesis of GC and pave the way for the development of an early diagnostic biomarker.

In this study, based on the high-throughput sequencing results from five paired GC samples, we identified the elevated expression of circAXIN1 in GC. The circRNA circAXIN1 encodes a novel protein, AXIN1-295aa. Here, we sought to determine how this novel protein interacts with the Wnt/β-catenin signaling pathway and what role it plays in GC.

## Materials and methods

### Tissue and cell culture

A total of 63 pairs of non-neoplastic gastric and GC tissue samples from patients who attended from Shenzhen Second People’s Hospital, China, were examined in this study. None of the tissues received any radiotherapy or chemotherapy prior to surgery, and they were stored in RNAlater (Thermo Fisher, Shanghai, China) immediately following surgery. All patients provided written informed consent, and the study was approved by the ethics committee of Shenzhen University School of Medicine. Immortalized human normal gastric epithelial cells (HFE-145) were obtained from Dr. Duane T. Smoot of Meharry Medical College, USA. The GC cell lines (AGS, MKN28) were obtained from the American Type Culture Collection (ATCC) and China Infrastructure of Cell Line Resources, respectively, while the GES-1, BGC-823, SGC7901, and NCI-N87(N87) cell lines were purchased from the Cell Bank of the Chinese Academy of Sciences (Shanghai, China). All cells were cultured in DMEM (Hyclone, Logan, Utah) with 10% FBS (Gibco) in an incubator at 37 °C with 5% CO_2_.

### Plasmids and cell transfection

The full length of circAXIN1-3xFLAG was chemically synthesized and cloned into vector pLC5-ciR using the EcoRI and BamHI sites. The pLC5-ciR vector contains artificial side flanking sequences and SA (splice acceptor)/SD (splice donor) sequences. The 3xFLAG was inserted before the stop codon of the putative open reading frame (ORF). The CMV-AXIN1-295aa linear overexpression vector served as a positive control. The mCherry-IRES-GFP was cloned into a psin-EF2 vector. The wild type and mutant internal ribosome entry sites (IRESs) (IRES115–257, IRES115–186, IRES187–257, IRES689–838, IRES689–763, and IRES764–838) were cloned into a P-Luc2-IRES-Report vector using Geneseed. (Guangzhou, China). Plasmids were transfected when cells reached a confluence of 30 to 50%, using Lipofectamine™ 3000 Transfection Reagent (Invitrogen, Shanghai, China). siRNAs for circAXIN1 knockdown were synthesized using Geneseed. (Guangzhou, China); the sequences were: si-hsa_circAXIN1_01 AGAGAGTTCAGGACAGATT; si-hsa_circAXIN1_02: GAGAGTTCAGGACAGATTG; and si-hsa_circAXIN1_03; AGAGTTCAGGACAGATTGA. Cells at 30 to 50% confluence were transfected with 60 nM siRNAs using Lipofectamine RNAiMAX (Invitrogen, Shanghai, China).

### RNA-sequencing assay

RNAs from five human GC samples and their adjacent normal tissues were sequenced on an Illumina Hiseq 2500 (Chi Biotech, Shenzhen, China). The reads were aligned to the human reference genome (version GRCh38), using the BWA aligner. Any circRNAs were identified using CIRI software (version 2). The identified circRNAs were then annotated with the gene annotation file corresponding to the reference genome and the full-length circRNA sequences were extracted. The full-length circRNA sequences for all circRNAs were considered to be the reference genome, and the fastq reads were mapped using the bowtie2 aligner. Then, count data were generated using bedtools multiBamCov with the bowtie2 output (converted to bam, sorted, and indexed). The count data were normalized (TPM, transcripts per kilobase million), and the R package limma was used to identify differentially expressed (DE) circRNAs. Fold-change values > 2 and *p*-values < 0.05 were considered to be the thresholds for defining significantly differentially expressed circRNAs.

### RT-PCR and real-time quantitative RT-PCR

Total RNA was extracted using Trizol reagent (251,808, Invitrogen), according to the manufacturer’s protocol. Nuclear and cytoplasmic RNAs were extracted using the Cytoplasmic & Nuclear RNA Purification Kit (Norgen Biotek). For circRNA detection, RNase R (10 U, Geneseed, was used for linear RNA digestion at 37 °C for 30 min. Then, RNA was recovered using the RNeasy MinElute Cleanup Kit (74,204, QIAGEN). Reverse transcription and real-time PCR were performed using GoScript™ Reverse Transcription Mix (A2800 and A6002, Promega). All primers were synthesized by Sangon Biotech; detailed information about the primers is shown in Supplementary Table 1. GAPDH and 18S were used as internal controls.

### Western blotting

Protein was extracted with 2x Laemmli sample buffer (Bio-Rad) with a protease inhibitor (Roche). Cytoplasmic and nuclear proteins were isolated using NEPER™ Nuclear and Cytoplasmic Extraction Reagents (Thermofisher Scientific). Western blotting was conducted as previously described [21]. The antibodies used were: FLAG (F1804, Sigma), Ubiquitin (ab7780, Abcam), HDAC (A0238, ABclonal), AXIN1 (#3323, Cell Signaling), AXIN1(NBP1–31013, Novus Biologicals), AXIN1 (A4747-01A, US biological), β-catenin (#8480, Cell Signaling), GSK3β (#12456, Cell Signaling), Rabbit IgG control (#3900, Cell Signaling), Mouse IgG control (#5415, Cell Signaling), GAPDH (#5174, Cell Signaling), and Wnt/β-Catenin Activated Targets Antibody Sampler Kit (#8655, Cell Signaling).

### Co-immunoprecipitation and mass spectrometry

Immunoprecipitation and co-immunoprecipitation were performed using a Pierce Classic Magnetic IP/Co-IP Kit (88,804, Thermo Fisher Scientific). Cells were lysed with cold lysis buffer and supernatant was collected after centrifugation at 13,000 g for 10 min. Approximately 1000 μg protein was incubated with specific IP antibody (1:50) at 4 °C on a rotating platform overnight. Pierce Protein A/G Magnetic Beads (25 μL) were added to the antigen sample/antibody mixture and incubated at room temperature for 1 h. After washing, the target antigen–antibody complex was eluted with 100 μL of Elution Buffer and 10 μL of Neutralization Buffer, followed by Western blotting analysis or mass spectrometry analysis at BGI (BGI, Shenzhen). VeriBlot for IP Detection Reagent (HRP) (ab131366, Abcam) was used to avoid the detection of heavy and light chains.

### Chromatin immunoprecipitation

A chromatin immunoprecipitation (ChIP) assay was performed using a Magna ChIP G kit (# 17–409, MAGNA0002, Millipore), according to the manufacturer’s instructions. After crosslinking and sonication, 50 μL of sheared DNA was incubated with 20 μL of protein G magnetic beads and anti-β-catenin (1:50, #8480, Cell Signaling) at 4 °C overnight. Rabbit (DA1E) mAb IgG XP Isotype Control (1:50, #3900, Cell Signaling) was used as a negative control. Then, protein/DNA complexes were eluted and free DNA was purified for the following qRT-PCR assay. Promoter primers for the detection of gene downstream of Wnt signaling (CD44, CMYC, C-Jun) were designed, and the amplified products were confirmed to contain the β-catenin TCF binding site 5′-A/T A/T CAAAG-3′. The primer sequences are shown in Supplementary Table 1. qRT-PCR was performed as previously described [21].

### Dual-luciferase reporter assay

Topflash and Fopflash reporters were obtained from Addgene (Cambridge, MA, USA). Two predicted IRES sequences and truncated sequences were inserted into the P-Luc2-IRES-Report vector (Geneseed, Guangzhou). Firefly and Renilla luciferase activity were measured using a Dual-Glo luciferase assay kit (Promega).

### Confocal immunostaining

Cells were inoculated in 35-mm petri dishes (NEST) and transfected with OV-circAXIN1 or circAXIN1 si1 for 48 h. Wnt agonist1 (S8178, Selleckchem) and XAV-939 (S1180, Selleckchem) (10 and 1 μM, respectively) were used as positive controls. After being fixed and permeabilized, cells were incubated with anti-β-catenin antibody (#2677, 1:100, Cell Signaling) at 4 °C overnight. The next day, cells were incubated with Alexa Fluor® 488-conjugated anti-rabbit secondary antibody (#4412, 1:500, Cell Signaling) for 1 h at room temperature in the dark. DAPI II (Abbott Molecular, Abbott Park, Illinois) was used to stain nuclei. Images were taken using a ZEISS confocal microscope.

### Immunohistochemistry

Immunohistochemical staining was performed using a DAB kit (ZLI-9017, ZSGB-BIO) and a Mouse Polymer kit (PV6002, ZSGB-BIO). Paraffin sections were first deparaffinized and rehydrated using a series of xylene and ethanol rinses. Slides were heated with EDTA repair solution (10 μM, ZLI-9067, ZSGB-BIO) for 20 min in a microwave oven. The slides were washed with PBS and allowed to cool to room temperature; they were then blocked with 3% hydrogen peroxide for 10 min. After thorough washing, the slides were incubated with a specific antibody (1:100) at 4 °C overnight. Ki-67 (GB111499, Servicebio, 1:1000), TCF-1 (#2203, Cell Signaling, 1:100), β-catenin (#8480, Cell Signaling, 1:100), c-Jun (#9165, Cell Signaling, 1:100), and Met (#8198, Cell Signaling, 1:100) antibodies were used. The next day, the slides were incubated with secondary antibody for 20 min at 37 °C. An equal volume of DAB was added for 10 min, for color rendering. After washing, hematoxylin was used as a counterstain and the slides were sealed using a neutral resin. The intensity scores of stained sections were assessed by two pathologists. The staining intensity was evaluated on a scale from 0 to 3 (0, negative; 1, weakly positive; 2, moderately positive; 3, strongly positive) and the percentage of positive cells was scored from 0 to 4 (0, negative; 1, 1–25% positive; 2, 26–50% positive; 3, 51–75% positive; 4, 76–100% positive). The final scores were calculated as percentage positive multiplied by staining intensity.

### Protein structure and prediction of protein–protein interactions

The amino acid sequence of AXIN1-295aa was predicted based on the ORF nucleotide sequence. According to a report in the literature [22], an SAMP (serine-alanine-methionine-proline) region, comprising 25 aa from APC, interacts directly with the regulators of the G protein signaling (RGS) domain of AXIN1. The AXIN1-295aa and APC SAMP structures were predicted by using the fold recognition method PHYRE [23] (http://www.sbg.bio.ic.ac.uk/~phyre2/html/page.cgi?id=index). ZDOCK [24] (http://zdock.umassmed.edu/) was used to predict the interaction between AXIN1-295aa and SAMP.

### In vitro proliferation, migration, invasion, and colony formation assays

Cell proliferation was determined using an EdU assay (Ribobio, Guangzhou). Following transfection for 48 h, cells were collected to conduct the assays, as previously described [25]. The cell proliferation rate was calculated by dividing the number of actively dividing cells (red) by the total number of cells (blue). Sterile Transwells® with 8.0-μm pore polycarbonate membrane inserts (3422 and 3428, Corning) were used to determine cell migration and invasion, respectively. After transfection for 48 h, AGS cells were preincubated with mitomycin-C (10 μg/mL, Sigma, St. Louis, MO) for 1 h at 37 °C for migration assay. N87 cells were not preincubated with mitomycin-C. A total of 5 × 10^4^ cells were seeded into the upper chamber without FBS. After incubation for 24 h at 37 °C, the cells were fixed and then stained with hematoxylin. The cells in five random fields were counted under a microscope. A wound healing assay was also performed to determine the migratory ability of the cells. Transfected cells were inoculated in 6-well plates and were preincubated with mitomycin-C (10 μg/mL, Sigma, St. Louis, MO) for 1 h at 37 °C. An artificial scratch was made, at time 0 h. The wound width from five random fields was measured at 0, 24, and 48 h. Cell viability was determined by a colony formation assay. Approximately 200 transfected cells were seeded in 6-well plates and their viability was determined after culturing for 2 weeks.. Each experiment was repeated three times.

### In vivo tumorigenesis and metastasis assays

Female BALB/c nude mice, aged 4 to 6 weeks, were obtained from Charles River Laboratories (Beijing, China) and used for the animal studies. All of the animal experiments were conducted in accordance with the principles of the Institutional Animal Care and Use Committee of Shenzhen University. The xenograft model was successfully established by injecting 5 × 10^6^ AGS cells into the right flank of the nude mice (*n* = 7 in each group). In the xenograft model group, cholesterol-conjugated circAXIN1-siRNA (10 nmol, Geneseed) was intratumorally injected twice a week for 3 weeks. Tumor volume was measured every 3 days, with the tumors then resected for subsequent experiments. For the in vivo lung metastasis assay, 1 × 10^6^ AGS cells were injected via the tail vein to assess tumor metastasis ability; circAXIN1-siRNA was injected via the tail vein twice a week for 6 weeks. The lung was resected and stained with hematoxylin and eosin (H&E) to count the cancer metastatic lesions.

### Statistical analysis

The Student’s *t*-test or one-way ANOVA was used to evaluate the significance of any differences. The data from three independent experiments are shown as means ±SD. A *p-value < 0.05 was considered statistically significant.

## Results

### Differential circRNA expression profile in human GC

To analyze the circRNA expression profile in GC and adjacent normal tissues, we performed circRNA sequencing of linear RNA-depleted RNA from five paired GC tissues and adjacent normal tissues. The RNAs from five human GC samples and their adjacent normal tissues were sequenced on an Illumina Hiseq 2500 system. The reads were aligned to the human reference genome (version GRCh38) using the BWA aligner. CircRNAs were identified using CIRI (version 2) software. The identified circRNAs were annotated using the gene annotation file corresponding to the reference genome, and the full-length circRNA sequences were then extracted. The full-length circRNA sequences for all circRNAs were considered to be the reference genome, and the fastq reads were mapped using the bowtie2 aligner. Then, count data were generated using bedtools multiBamCov with the bowtie2 output (converted to bam, sorted, and indexed). The count data were normalized (TPM) and the R package limma was used to identify differentially expressed (DE) circRNAs. Fold-change values > 2 and *p*-values < 0.05 were considered to be the thresholds for defining significant DE circRNAs.

**Fig. 1:**
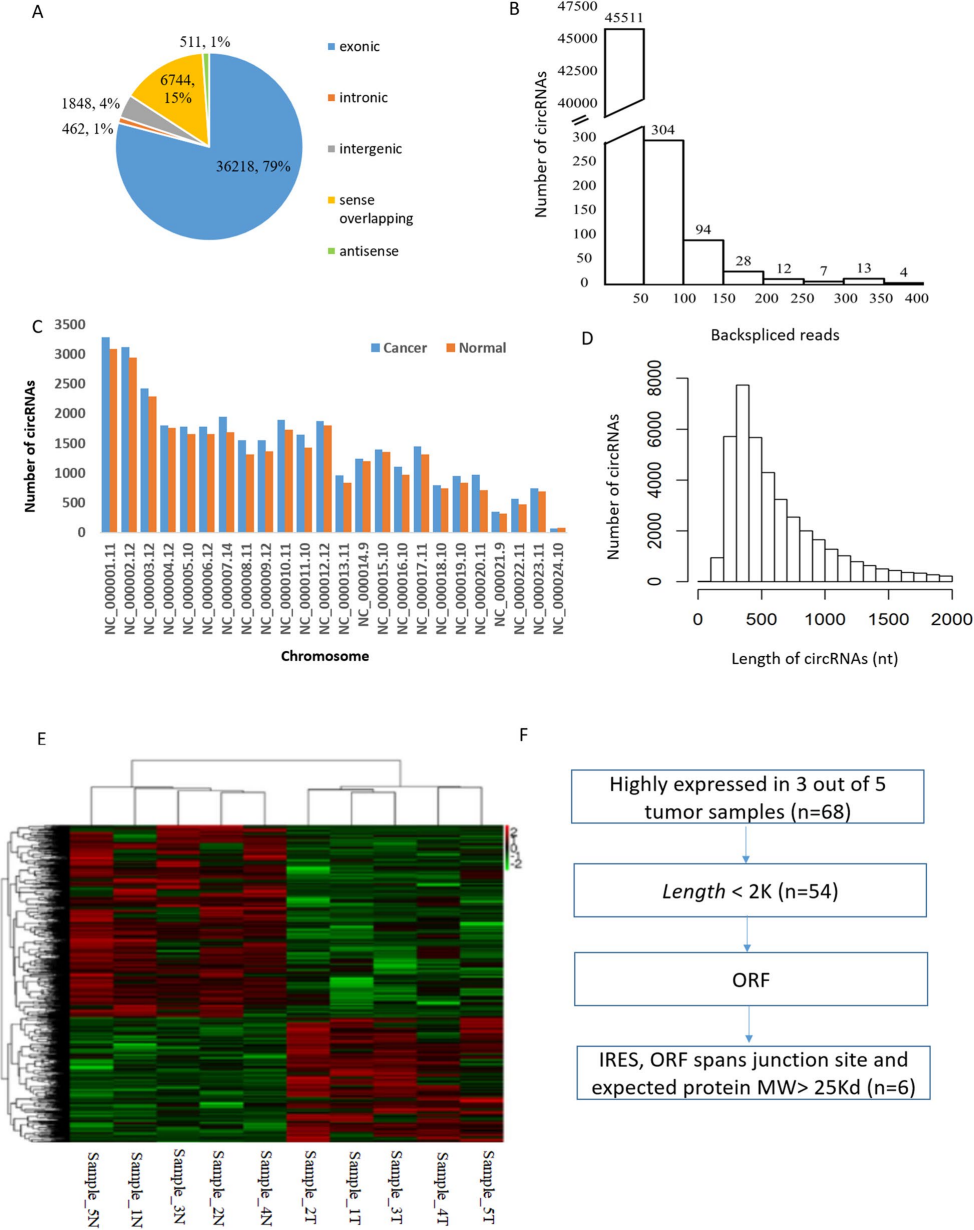
Differential expression profile of circRNAs in human gastric cancer (GC) and adjacent normal tissues. **a** The numbers and ratios of circRNAs originating from different regions of the genome. **b** The number of circRNAs and back-spliced reads identified. X-axis: the backspliced reads of circRNAs detected in this study. Y-axis: the number of circRNAs. **c** The chromosome distribution pattern of circRNAs shows no difference between the cancer and normal groups. **d** The length of the majority of the circRNAs is less than 1500 nucleotides (nt). **e** The dendrogram shows the relationship between the samples and the differentially expressed circRNAs. The differential expression in the normal and cancer samples was clearly distinguishable. **f** The workflow used to identify functional circRNAs with coding potential in GC

A total of 45,783 circRNAs were identified from all the samples. Of the total number of circRNAs, 79% (36,218/45,783) were exonic, 1% (462/45,783) were intronic, 4% (1848/45,783) were intergenic, 15% (6744/45,783) were sense overlapping, and 1% (511/45,783) were antisense (Fig. 1a). The number of back-spliced reads for the majority of the circRNAs was less than 100 (Fig. 1b). The distribution of circRNAs among chromosomes was heterogeneous, with most circRNAs in chromosome NC_000001.11 (chromosome 1) (Fig. 1c). No differences were observed between the chromosome distribution patterns of the circRNAs in the cancer and the normal groups. The lengths of the majority of the circRNAs were less than 1500 nucleotides (nt) (Fig. 1d). The dendrogram shows the relationship between the samples and the differentially expressed circRNAs. The circRNA expression in the normal and cancer samples is clearly distinguishable (Fig. 1e).

### CircAXIN1 encodes the novel protein AXIN1-295aa

**Fig. 2:**
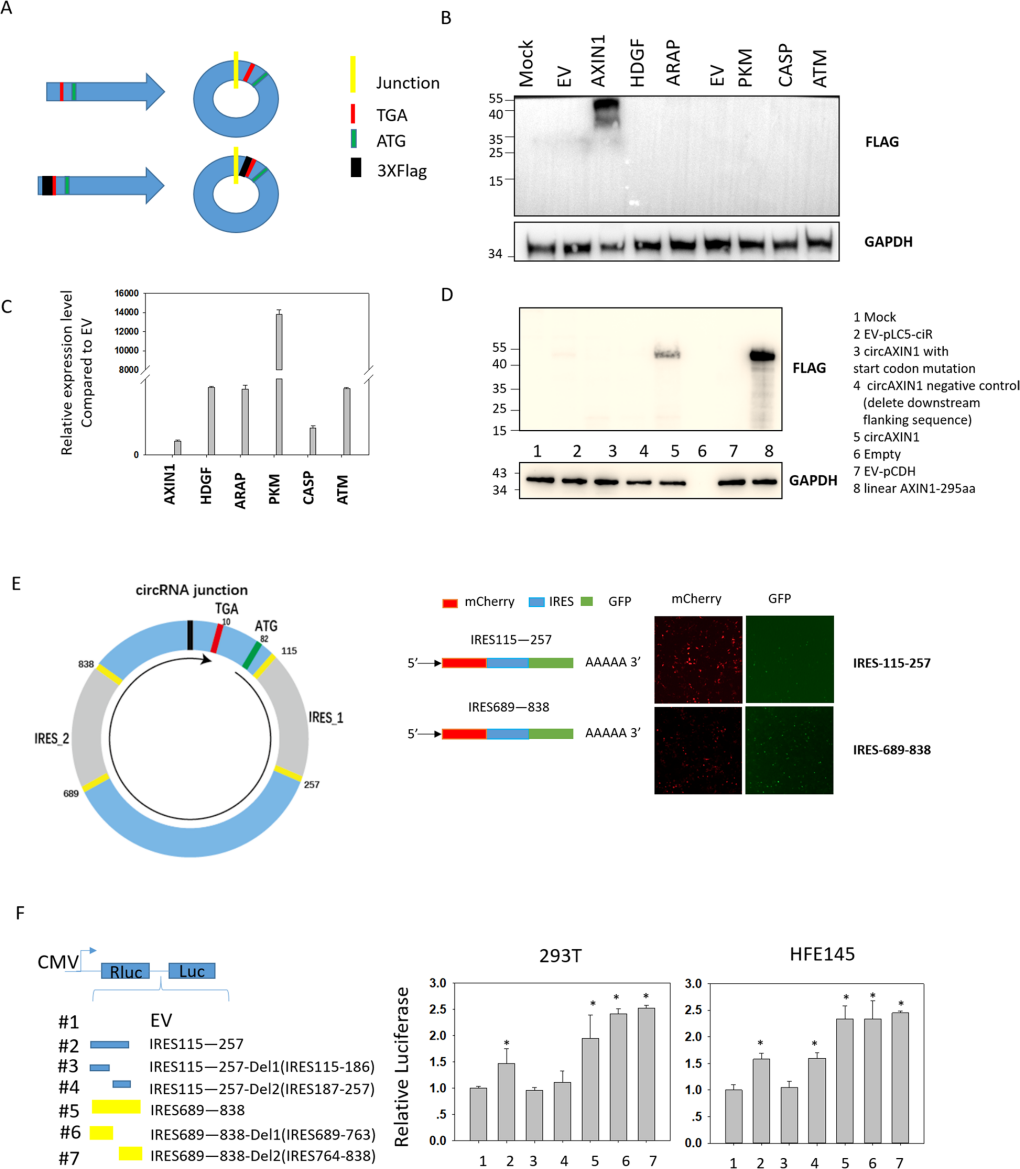
CircAXIN1 encodes a novel protein AXIN1-295aa. **a** An illustration showing the construction of FLAG-tagged circRNA. **b** CircAXIN1 potentially encodes a novel protein, AXIN1-295aa. Immunoblotting of lysates from cells transfected with FLAG-tagged circRNAs. **c** The circRNAs were successfully overexpressed as determined by quantitative real-time PCR using divergent primers. **d** AXIN1-295aa is encoded by the circularized RNA, circAXIN1. The plasmids with start codon mutations and with deletion of downstream flanking sequences were unable to generate AXIN1-295aa. The linearized circAXIN1 ORF was cloned into a CMV-induced expression vector (linear AXIN1-295aa) to serve as a positive control. **e** An illustration of the IRESs and ORF of circAXIN1. Both IRESs are active. The IRES was cloned between mCherry and GFP, as shown. The constructs were transfected into 293 T cells and images were taken to show the expression of mCherry and GFP. **f** CircAXIN1 has two active IRESs. The two IRESs and partial IRESs were constructed between tandem Rluc and Luc reporters with independent start and stop codons. The constructs were transfected into cells. The relative luciferase activity was determined

Based on the circRNA sequencing data from five paired GC samples, we found 68 circRNAs that were highly expressed in at least three samples (Supplementary Table 2 and Fig. 1f). Of these, six circRNAs were selected based on their length, ORF, ORF position, and IRES. We then constructed FLAG-tagged circRNA overexpression plasmids by inserting a FLAG tag before the stop codon, so that the junction site and the ORF remained intact (Fig. 2a). Immunoblotting analysis after transfection of the six circRNA overexpression constructs in 293 T cells revealed that circAXIN1 may encode a novel protein (Fig. 2b). CircAXIN1 is located at chr16: 396,147–397,106. It is composed of exon2 from the parental gene AXIN1 and is 959 nt in length. As the protein was predicted to have 295 amino acids, we named it AXIN1-295aa. To rule out the possibility that the lack of protein expression was due to low transfection efficiency, we performed qPCR using a specific divergent primer for each construct. The transfection efficiency was determined to be high for all six constructs (Fig. 2c). To confirm that the novel protein was truly encoded by circAXIN1, we constructed deletion and mutation plasmids, which showed that mutating the start codon or deleting the downstream flanking sequence of the overexpressed plasmid led to a lack of protein expression. This indicates that expression of the novel protein requires a complete ORF and correct circularization of circRNA (Fig. 2d). In addition, we constructed a plasmid expressing linear AXIN1-295aa. The molecular weights of AXIN1-295aa expressed from circRNA (lane 5) and from the linear construct (lane 8) were the same, which suggests that AXIN1-295aa was expressed as we predicted based on the ORF and nucleotide sequence information (Fig. 2d). Next, we investigated the presence of IRESs on circAXIN1 and identified two potential IRESs: IRESs 115–257 and 689–838 (Fig. 2e). These IRESs were cloned downstream of mCherry and upstream of GFP. As shown in Fig. 2e, both IRESs were active and induced the expression of GFP. The second IRES, 689–838, was evidently more active than the first IRES, 115–257, as more GFP was expressed, while mCherry was expressed at a lower level by the second IRES. Based on the GFP results, we conducted a dual-luciferase assay to verify the IRES activity. As shown in Fig. 2f, both IRESs were active in two cell lines (293 T and HFE-145 cells), with greater activity from IRES 689–838. Interestingly, it seemed that IRES 115–257 required a complete sequence to activate translation, whereas IRES 689–838 needed just half of the sequence.

### CircAXIN1 is highly expressed and positively associated with lymph node metastasis in GC

**Fig. 3:**
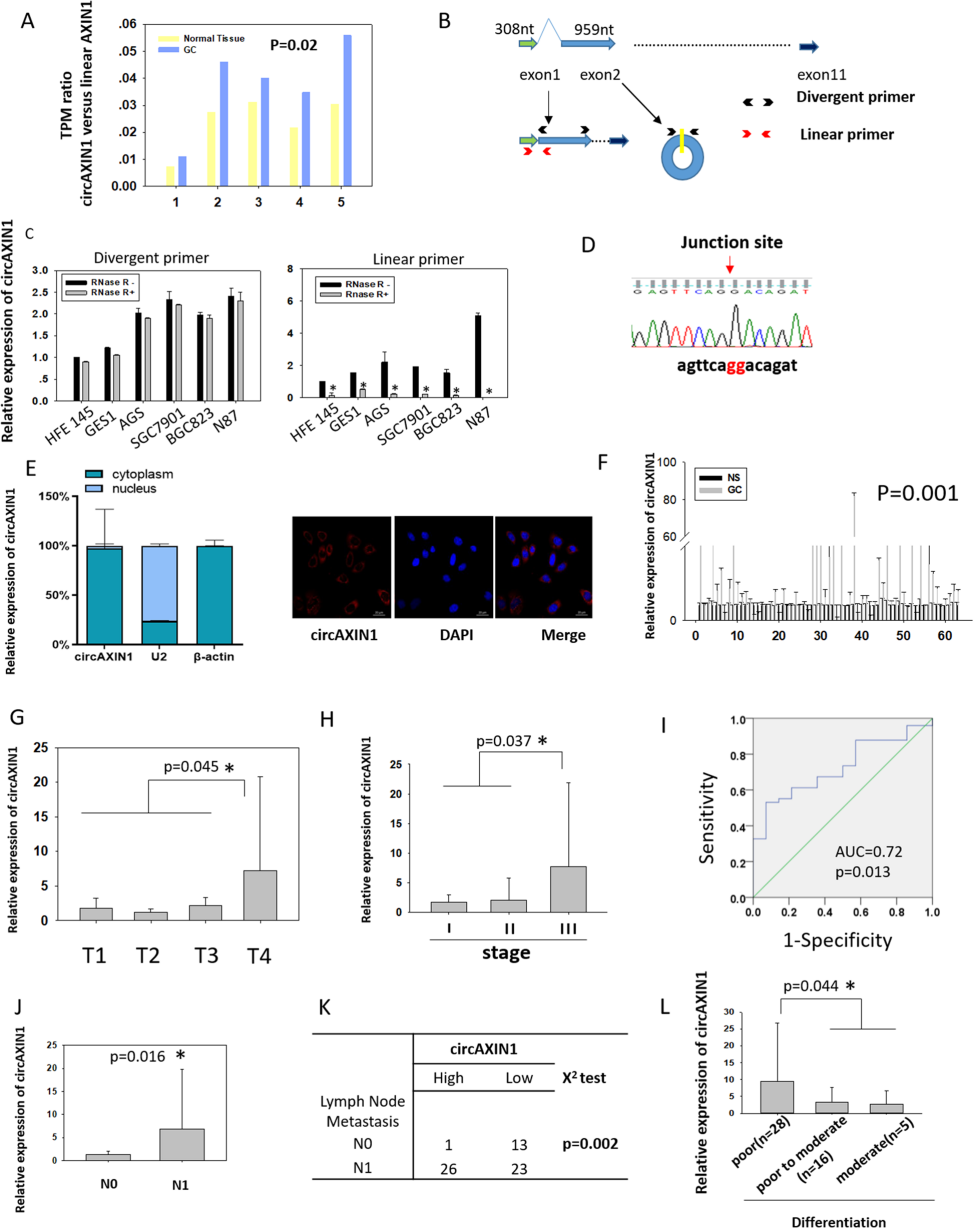
Characterization of circAXIN1. **a** The TPM ratio of circAXIN1 versus linear AXIN1 is elevated in GC samples obtained from a cohort of patients in the ATGC database. **b** An illustration of the linear AXIN1 and circAXIN1 genomic regions. CircAXIN1 is formed from the head to tail splicing of exon2 of the AXIN1 parental gene. The specific divergent and convergent primers were designed as shown to detect circAXIN1 and linear AXIN1, respectively. **c** CircAXIN1 is expressed at a higher level in GC cells compared with that in normal gastric epithelial cells. CircAXIN1 is resistant to RNase R-digestion but linear AXIN1 is not. **d** Sanger sequencing was performed to validate the back splicing of circAXIN1 using divergent primers. **e** CircAXIN1 is mostly localized in the cytoplasm. Left: cytoplasmic and nuclear RNA was extracted, and quantitative PCR was performed to detect the relative subcellular localization of circAXIN1. U2 was used as the positive control for nuclear localization and β-actin for cytoplasmic localization. Right: Using probes specific to the junction site, FISH indicated that circAXIN1 is localized in the cytoplasm. **f** CircAXIN1 is highly expressed in GC tissues (*p* = 0.001). NS: Normal Sample. **g** CircAXIN1 is highly expressed in T4 compared with its expression in T1–3. **h** CircAXIN1 is overexpressed in stage III compared with its expression in stage I and II. **i** Receiver operating characteristic (ROC) curve analysis of normalized circAXIN1 expression of GC with and without lymph node metastasis. The area under the ROC curve (AUC) conveys the accuracy, in terms of its sensitivity and specificity, of this biomarker in distinguishing GC lymph node metastasis. **j** CircAXIN1 is significantly highly expressed in GC with lymph node metastasis. **k** The expression of circAXIN1 is positively associated with lymph node metastasis. **l** CircAXIN1 is particularly highly expressed in poorly differentiated tumors compared to moderately differentiated and moderately to poorly differentiated tumors with lymph node metastasis

To characterize circAXIN1, we analyzed the TPM values of circAXIN1 and linear AXIN1 genes in five samples from GC patients from TCGA (BioProject: PRJNA638934) (Supplementary Fig. 1a and b). Both expression was increased in GC. As shown in Fig. 3a, the TPM ratio of circAXIN1 versus linear AXIN1 was around 0.01 to 0.06 and was elevated in all five paired GC samples, suggesting that circAXIN1 was expressed at a relatively high level. We also designed divergent primers specific for circAXIN1 and for linear AXIN1 mRNA (Fig. 3b). The quantitative PCR results revealed that circAXIN1 was highly expressed in GC cell lines such as AGS, SGC 7901, BGC823, and N87. The expression of circAXIN1 changed very little in RNase R-treated samples from different cell lines, while the linear AXIN1 mRNA was degraded upon enzyme digestion (Fig. 3c). The PCR product was sequenced, and we determined that PCR using the divergent primer correctly amplified the junction site of circAXIN1 (Fig. 3d). We then performed nuclear and cytoplasmic RNA extraction combined with PCR and fluorescence in situ hybridization (FISH) to determine the distribution of circAXIN1. We found circAXIN1 to be mostly localized in the cytoplasm (Fig. 3e), which suggests that it might be translated.

We also found circAXIN1 to be significantly highly expressed in 63 paired GC samples (*p* = 0.001) (Fig. 3f). The expression level of circAXIN1 was significantly higher in T4 compared with its expression in T1–3 (*p* = 0.045), suggesting that the expression of circAXIN1 is associated with tumor invasion depth (Fig. 3g). Based on AJCC (American Joint Committee on Cancer) staging, the expression of circAXIN1 was significantly higher in stage III versus stage I and II (*p* = 0.037), suggesting that circAXIN1 is related to tumor progression (Fig. 3h). The receiver operating characteristic (ROC) curve yielded an area under the curve (AUC) = 0.72, *p* = 0.013 (Fig. 3i) and a cutoff value of 1.899 in predicting lymph node metastasis. The expression level of circAXIN1 was found to be further enhanced in tumors with lymph node metastasis (Fig. 3j, *p* = 0.016), and a positive association was revealed between the expression of circAXIN1 and lymph node metastasis (Fig. 3k, *p* = 0.002). We further analyzed the expression profile of circAXIN1 in GC with lymph node metastasis. This showed that circAXIN1 was particularly highly expressed in poorly differentiated tumors but not in moderately and poorly to moderately differentiated tumors, suggesting that high expression of circAXIN1 is linked with poor differentiation (Fig. 3l). Taking these results together, we can conclude that circAXIN1 was highly expressed in GC, especially in advanced tumors, and was positively related with tumor invasion depth and lymph node metastasis, which suggests it might be a useful prognostic factor in GC.

### Characterization of AXIN1-295aa

**Fig. 4:**
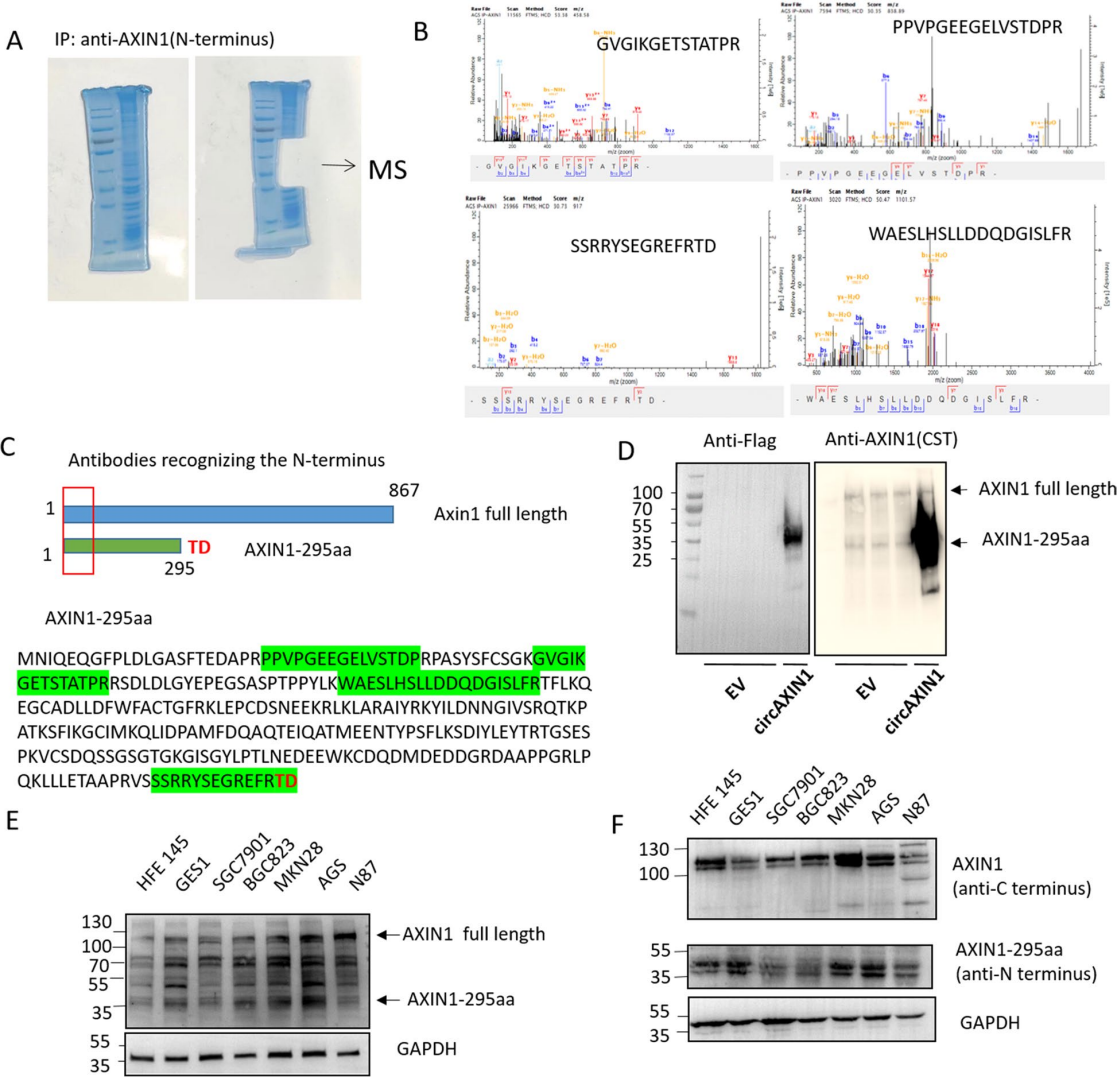
Characterization of AXIN1-295aa. **a** An IP assay was conducted to detect endogenous AXIN1-295aa using an anti-AXIN1 N-terminus antibody. Mass spectrometry was performed using the gel cut from 25 to 55 kDa following SDS-PAGE. **b** The recognized peptides from mass spectrometry match with AXIN1-295aa. The sequences are highlighted in green in **c**. **c** AXIN1-295aa is homologous to the N-terminus of full-length AXIN1 with two aa differences at position 294 to 295 aa. Two amino acids, “TD”, of circAXIN1, which were distinguishable from full-length AXIN1, were detected. Commercial antibodies recognizing the N-terminus of AXIN1 are available. **d** The antibody that recognizes the N-terminus of AXIN1 was able to detect endogenously and ectopically expressed AXIN1-295aa. Left: anti-FLAG antibody was used in immunoblotting to detect overexpressed AXIN1-295aa. Right: AXIN1 antibody that can recognize the N-terminus of AXIN1 detected both full-length AXIN1 as well as endogenous and overexpressed AXIN1-295aa. **e** Full-length AXIN1 and AXIN1-295aa are detected in GC cell lines by an anti-AXIN1 N-terminus antibody (CST). **f** Full-length AXIN1 is detected using an anti-C-terminus antibody and AXIN1-295aa is detected using an anti-N-terminus antibody (US Biological), respectively, in GC cell lines

To characterize AXIN1-295aa, we performed mass spectrometry (MS) following the immunoprecipitation and overexpression of FLAG-tagged circAXIN1. First, we performed immunoblotting after the immunoprecipitation of FLAG to confirm the expression of the FLAG-tagged novel protein and its successful immunoprecipitation by the anti-FLAG antibody. Following SDS-PAGE of the lysate from the immunoblotting process, gels with molecular weights ranging from 25 to 55 kDa were sent for MS analysis (Supplementary Fig. 2a), and the results were found to align with the peptide encoded by circAXIN1, as illustrated in Supplementary Fig. 2b and highlighted in Supplementary Fig. 2c. The full size of AXIN1 is approximately 110 kDa, whereas the sizes of the gels we analyzed ranged from 25 to 55 kDa, which suggests that the peptides detected were not from AXIN1 but from circAXIN1. To further confirm our hypothesis, we obtained antibodies that recognize the N-terminus of AXIN1, provided by the commercial suppliers CST (AXIN1 (C7B12) rabbit mAb #3323) and US Biological (A4747-01A). We also performed the MS analysis after IP using antibody that recognizes the N-terminus of AXIN1 (A4747-01A, US Biological) without any transfection to detect the endogenously expressed AXIN1-295aa. Similarly, gels cut between 25 to 55 kDa were analyzed. As shown in Fig. 4b and c, several aa sequences aligned to AXIN1-295aa were detected by MS, especially the specific aa sequence “SSRRYSEGREFRTD”, which is encoded by circAXIN1 but not AXIN1. This further confirms the existence of endogenously expressed AXIN1-295aa.

A FLAG-tagged overexpression plasmid was transfected into 293 T cells, and the same lysate was used to blot FLAG as that used in the immunoblotting of AXIN1. The AXIN1 antibody (CST) could not only recognize the full length of AXIN1 but also detect the overexpressed AXIN1-295aa that was recognized by the anti-FLAG antibody. Furthermore, the AXIN1 antibody could detect the endogenously expressed AXIN1-295aa (lanes 1–3) and the full-length AXIN1 (lanes1–4 upper band) (Fig. 4d), which strongly suggests that AXIN1-295aa is encoded by circAXIN1. Using the CST antibody, we were able to detect AXIN1-295aa in GC cell lines. We found AXIN1-295aa to be highly expressed in the GC cell lines MKN 28 and AGS, as compared with its expression in the normal gastric epithelial cell line HFE145 (Fig. 4e). More importantly, AXIN1-295aa is expressed at a comparative level to that of full-length AXIN1 in several GC cell lines, suggesting that circAXIN1 is truly translated. We obtained similar results using another N-terminus-recognizing antibody (US Biological) to detect the expression level of AXIN1-295aa and a C-terminus-recognizing antibody to observe the full length of AXIN1 (Fig. 4f).

### CircAXIN1 plays an oncogenic role in GC via AXIN1-295aa

**Fig. 5:**
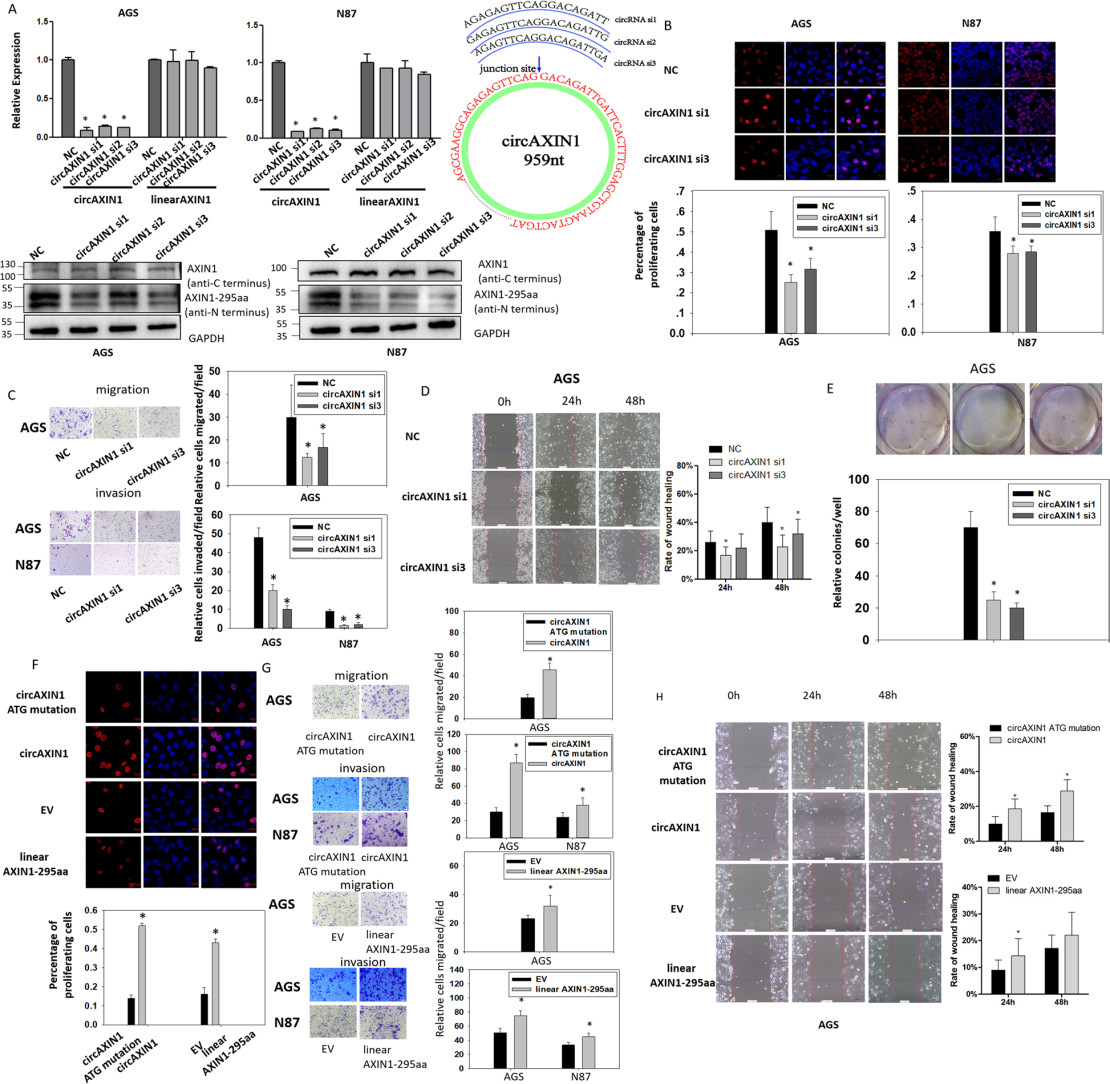
The biological function of circAXIN1. **a** The RNA and protein levels of circAXIN1 are significantly knocked down by three specific siRNAs, while the expression of linear AXIN1 is unaffected. An illustration of the junction site of circAXIN1 targeted by siRNAs is shown. **b** Cell proliferation is significantly repressed by circAXIN1 siRNA1 and siRNA3, in both AGS and N87 cells in the EdU assay. **c** Cell migration of AGS (treated with 10 μg/mL mitomycin for 1 h) and invasion of AGS and N87 cells are both inhibited by circAXIN1 siRNA transfection. **d** Cell migration is inhibited by circAXIN1 siRNAs in AGS cells (treated with 10 μg/mL mitomycin for 1 h) in the wound healing assay. **e** Colony formation ability is suppressed in AGS cells with circAXIN1 siRNA transfection. **f-h** Cell proliferation (**f**), cell invasion (**g**), and cell migration (**g-h**) are enhanced with circAXIN1 or linear AXIN1-295aa transfection in AGS and N87 cells. AGS cells are treated with 10 μg/mL mitomycin for 1 h in a migration assay, 48 h after transfection (**g-h**). EV, empty vector

To investigate the role played by circAXIN1 in the development of GC, we designed siRNAs against circAXIN1 based on its junction site sequences. As shown in Fig. 5a, three siRNAs successfully knocked down the expression of circAXIN1, but not the mRNA of linear AXIN1. siRNA also caused downregulation of AXIN1-295aa but not full-length AXIN1 (Fig. 5b) in AGS and N87 cells. In the EdU assay, the proliferation of the circAXIN1 siRNA-transfected AGS and N87 cells was retarded compared with that of the control (Fig. 5b). The downregulation of circAXIN1 resulted in reduced cell migration in AGS cells pretreated with mitomycin, as revealed by the transwell and wound healing assays, suggesting that the reduced migration is independent of proliferation (Fig. 5c upper panel and 5d, respectively). Similar results were obtained in N87 cells (Supplementary Fig. 3a). In addition, the suppression of circAXIN1 led to decreased invasive ability (Fig. 5c lower panel) and colony formation capacity (Fig. 5e) in GC cells.

Conversely, the overexpression of AXIN1-295aa was achieved by the use of either circAXIN1 or linear AXIN1-295aa. Compared with circAXIN1 ATG mutant-transfected cells, cells transfected with circAXIN1 displayed an increased proliferation rate. Similarly, linear AXIN1-295aa overexpression also resulted in enhanced proliferation in AGS cells (Fig. 5f). The enforced expression of circAXIN1 and linear AXIN1-295aa was observed to promote migration and invasion in AGS and N87 cells (Fig. 5g and h and Supplementary Fig. 3b and c).

**Fig. 6:**
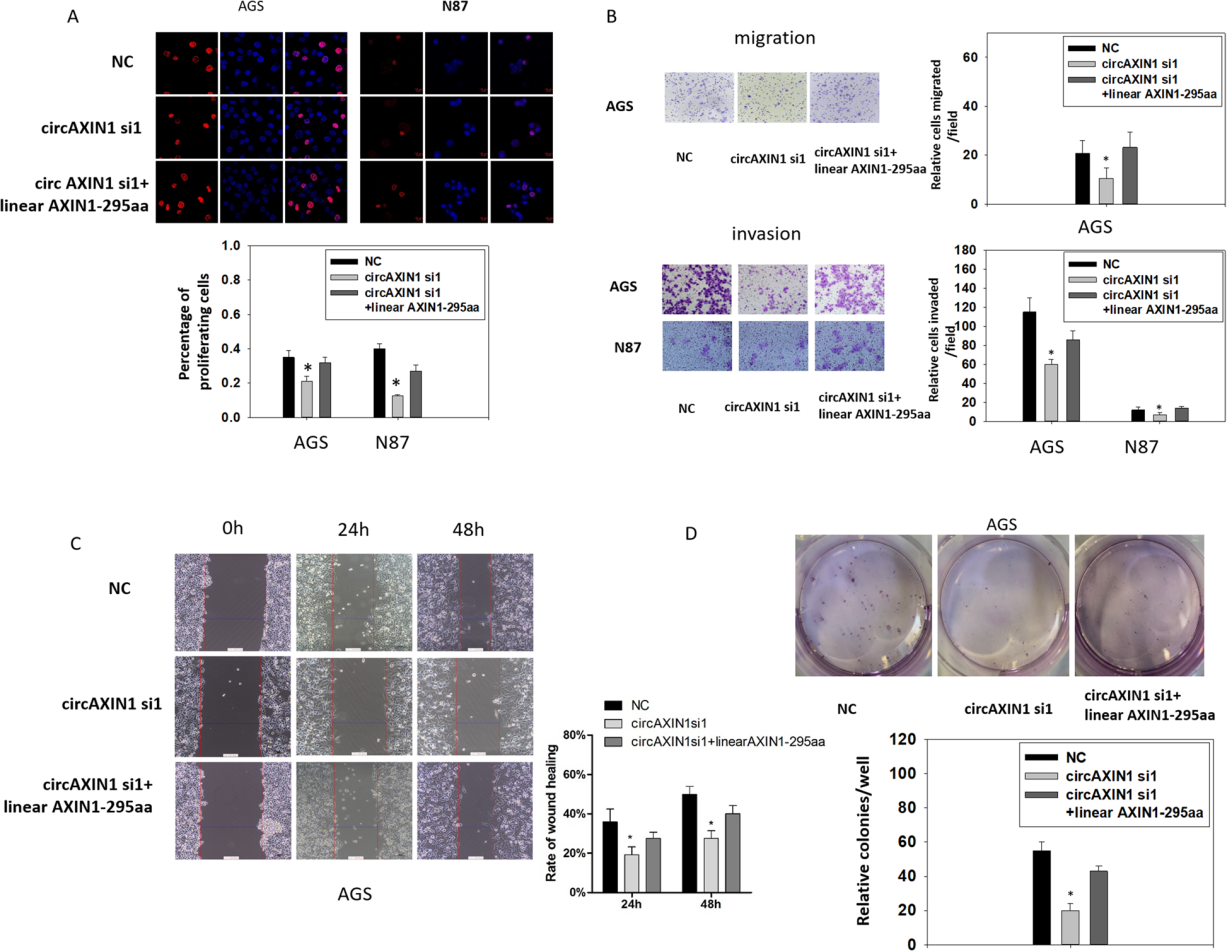
The inhibitory effect of circAXIN1 siRNA is rescued by linear AXIN1-295aa. **a** Cell proliferation of AGS and N87 is inhibited by circAXIN1 siRNA and reversed by linear AXIN1-295aa transfection. **b** Cell migration of AGS (treated with 10 μg/mL mitomycin for 1 h) and invasion of AGS and N87 are both inhibited by circAXIN1 siRNA and rescued with linear AXIN1-295aa transfection. **c** The inhibitory effect of circAXIN1 siRNA on cell migration is reversed by linear AXIN1-295aa. AGS cells are treated with 10 μg/mL mitomycin for 1 h in the wound healing assay, 48 h after transfection. **d** Colony formation ability is suppressed in AGS cells by circAXIN1 siRNA and rescued by linear AXIN1-295aa transfection

To identify the biological role of AXIN1-295aa independent of circAXIN1, we first knocked down the expression of circAXIN1 and replenished the expression of AXIN1-295aa by cotransfecting the plasmid expressing AXIN1-295aa in a linear format. As shown in Fig. 6a, the proliferation of AGS and N87 cells was inhibited by repressing circAXIN1 and restored by the additional expression of linear AXIN1-295aa. Similarly, the migration and invasion of AGS and N87 cells were halted by blocking circAXIN1, whereas the inhibitory effect was relieved by linear AXIN1-295aa (Fig. 6b and Supplementary Fig. 3d). In the wound healing assay, linear AXIN1-295aa expression enhanced cell migration, which was inhibited by circAXIN1 siRNA, which implies that AXIN1-295aa mediated the migration-promoting role of circAXIN1 (Fig. 6c). Linear AXIN1-295aa expression also restored the reduction in colony formation ability caused by the inhibition of circAXIN1 (Fig. 6d). Taking these results together, we can conclude that circAXIN1 plays an oncogenic role in GC via AXIN1-295aa.

### AXIN1-295aa competitively binds to APC

**Fig. 7:**
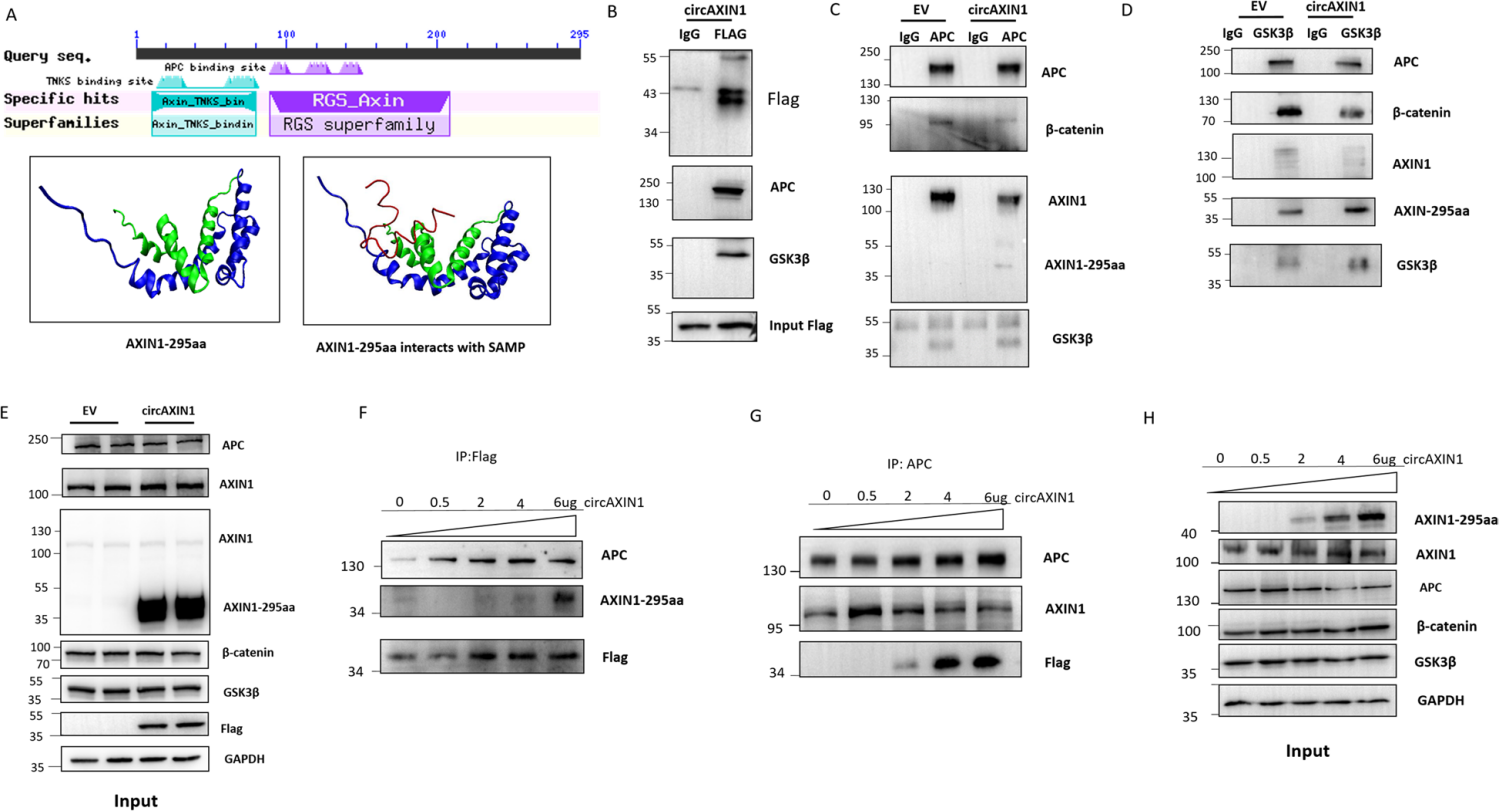
AXIN1-295aa competitively binds to APC. **a** Upper panel: Sequence alignment reveals that AXIN1-295aa contains an RGS domain. Lower panels: The 3D structure shows the simulated binding interface between AXIN1-295aa and the SAMP structure of APC, created using the online software packages PHYRE and ZDOCK. The left image shows the predicted 3D structure of AXIN1-295aa. The red line is the SAMP domain of APC, which is the domain that interacts with full length AXIN1. The right image illustrates the potential interaction interface between the SAMP domain of APC and AXIN1-295aa. **b-d** A co-IP assay was performed to evaluate the binding potential among AXIN1-295aa, AXIN1, APC, β-catenin, and GSK3β. **b** AXIN1-295aa binds to APC and GSK3β. **c** Overexpression of circAXIN1 attenuates the binding between APC with AXIN1 and β-catenin. **d** Overexpression of circAXIN1 attenuates the binding between GSK3β and β-catenin. **e** The expression of APC, AXIN1, β-catenin, and GSK3β is unchanged after transfection of circAXIN1 in 293 T cells. **f-g** AXIN1-295aa competitively binds to APC. **f** Various doses of circAXIN1 were transfected into 293 T cells. IP using FLAG antibody was performed to detect the change in interaction between APC and AXIN1-295aa. **g** IP was performed using anti-APC antibody. Binding of APC to AXIN1 is weakened and binding of APC to AXIN1-295aa is enhanced with higher doses of circAXIN1 transfection. **h** The expression of AXIN1-295aa increased in a dose-dependent manner. The expression of AXIN1, APC, GSK3β, and β-catenin barely changed with the various doses of circAXIN1 transfection

The predicted amino acid sequence of AXIN1-295aa shares 98% homology with the parental protein AXIN1, which contains all of the RGS domain, the region that interacts with APC (Fig. 7a), but not with β-catenin. We predicted the AXIN1-295aa structure using the fold recognition method PHYRE [23]. The protein contained two domains, i.e., the tankyrase-binding N-terminus segment of AXIN (TKNS), shown in blue, and the RGS domain, shown in green (Fig. 7a, lower left). As previously reported [22], the SAMP region, comprising 25 amino acids from APC, interacts directly with the RGS of AXIN1. Subsequently, we used ZDOCK [24] to predict the interaction between AXIN1-295aa and SAMP (shown as the red line in Fig. 7a). As shown in Fig. 7a (lower right), the RGS domain from AXIN1-295aa interacted with SAMP, which strongly suggests that AXIN1-295aa competes with AXIN1 to bind APC. AXIN1-295aa might function as a dominant negative for AXIN1.

Next, we overexpressed FLAG-tagged circAXIN1 in 293 T cells and pulled down the FLAG using a specific anti-FLAG antibody and the immunoblotted APC and GSK3β. The results showed that AXIN1-295aa interacted with APC and GSK3β (Fig. 7b). To characterize the effect the interaction between APC, β-catenin, AXIN1, and GSK3β has upon the overexpression of circAXIN1, we conducted a co-immunoprecipitation (co-IP) assay. The results showed that APC bound smaller quantities of β-catenin and AXIN1 when circAXIN1 was overexpressed, but bound larger quantities of AXIN1-295aa, which suggests that AXIN1-295aa competes with AXIN1 to bind to APC (Fig. 7c). The interaction between APC and GSK3β changed very little. The interaction between GSK3β and β-catenin decreased and that between GSK3β and AXIN1-295aa increased under circAXIN1 overexpression (Fig. 7d). Thus, we can infer that AXIN1-295aa competitively interacted with APC and GSK3β in the destruction complex. To rule out the possibility that the increased or decreased interaction was the result of a change in gene expression, we used the cell lysate before co-IP to immunoblot the components of the destruction complex. Although we observed no significant changes in the expression levels of APC, GSK3β, AXIN1, or β-catenin, the transfection of circAXIN1 successfully expressed AXIN1-295aa, as revealed by the immunoblotting method using the AXIN1 antibody that recognized the N-terminus of AXIN1 and the FLAG antibody (Fig. 7e).

To further confirm the competitive binding between AXIN1-295aa and AXIN1 toward APC, we achieved increasing doses of AXIN1-295aa overexpression and performed the co-IP of AXIN1-295aa and APC. The results showed that more APC was pulled down by increasing the overexpression of AXIN1-295aa (Fig. 7f). Meanwhile, less AXIN1 and more AXIN1-295aa were observed to bind to APC with increases in the overexpression of AXIN1-295aa, indicating competitive binding of APC between AXIN1-295aa and AXIN1 (Fig. 7g). The total lysates were analyzed to ensure the overexpression of AXIN1-295aa in a dose-dependent manner and the absence of an expression change in each component of the destruction complex (Fig. 7h).

### AXIN1-295aa activates the Wnt/β-catenin signaling pathway

**Fig. 8:**
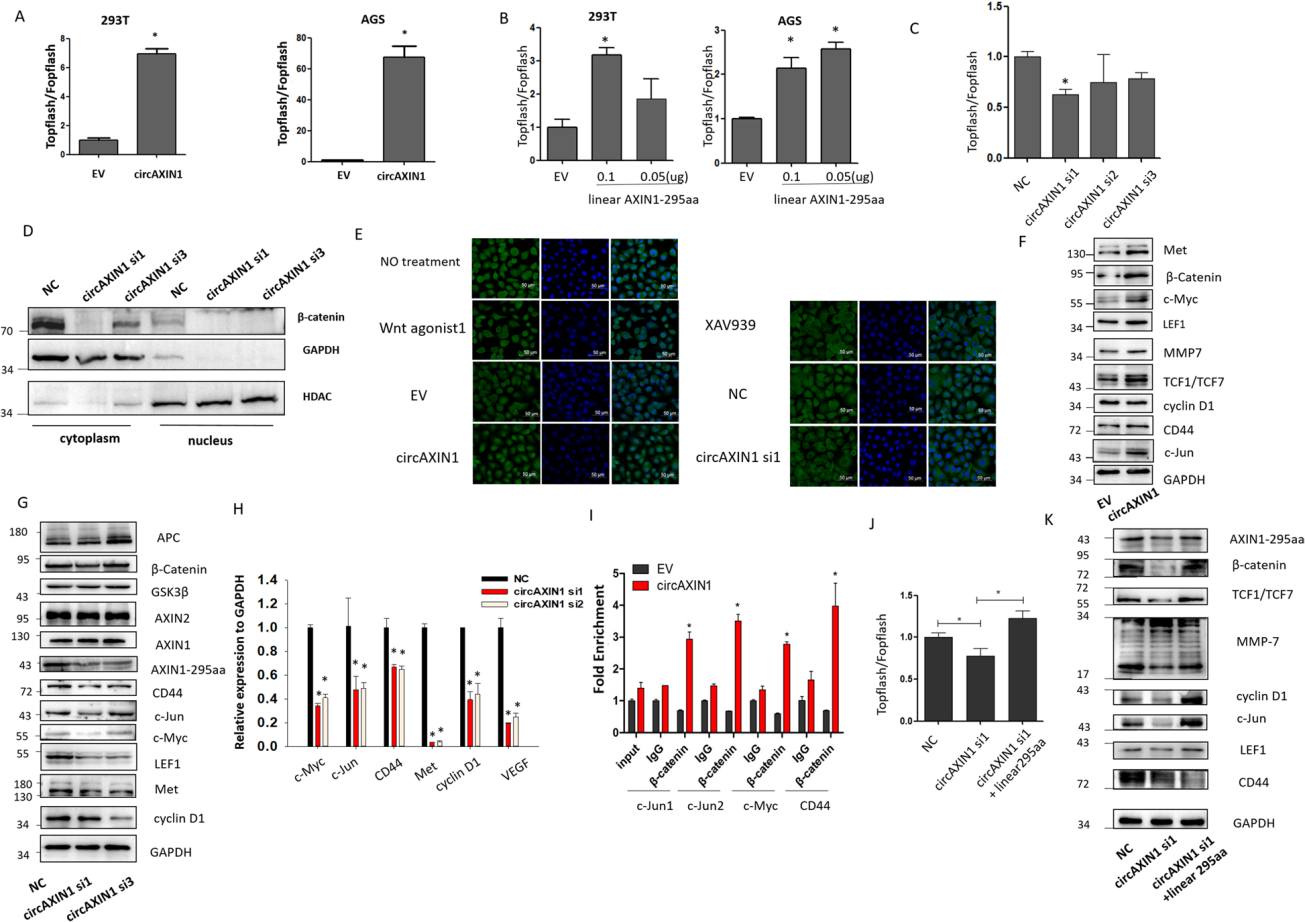
CircAXIN1 activates the Wnt/β-catenin signaling pathway. **a-b** CircAXIN1 and AXIN1-295aa activate Wnt/β-catenin-dependent transcription. **a** Topflash/Fopflash assays were performed in 293 T and AGS cells with circAXIN1 transfection. **b** Topflash/Fopflash assays were performed in 293 T and AGS cells with linear-295aa transfection. **c** Topflash/Fopflash assays were performed in 293 T cells with individual circAXIN1 siRNA transfection. **d** Inhibition of circAXIN1 causes downregulation of β-catenin in both the cytoplasm and nucleus. Expression of β-catenin in the cytoplasm and nucleus with individual circAXIN1 siRNA transfection. **e** CircAXIN1 overexpression enhances β-catenin nuclear translocation and circAXIN1 siRNA inhibits β-catenin nuclear translocation. Immunofluorescence localization of β-catenin was detected with circAXIN1 or circAXIN1 siRNA transfection. Wnt agonist1 and XAV939 (a Wnt/β-catenin signaling inhibitor) served as positive controls. **f** CircAXIN1 overexpression activates the downstream gene expression of the Wnt/β-catenin pathway. **g** Individual circAXIN1 siRNA transfection leads to the reduced expression of Wnt/β-catenin pathway downstream genes. **h** The mRNA levels of c-Myc, c-Jun, CD44, Met, cyclinD1, and VEGF decrease with each circAXIN1 siRNA transfection. **i** More β-catenin binds to the TCF-dependent promoter of Wnt/β-catenin downstream genes with overexpression of circAXIN1. A ChIP assay was performed using anti-β-catenin to detect the binding of β-catenin to the promoters of *c-Jun*, *c-Myc*, and *CD44*. C-Jun1/2 means region1 or 2 of the c-Jun promoter. **j** Linear AXIN1-295aa rescues the inhibitory effect of circAXIN1 siRNA on Topflash activity. Topflash/Fopflash assays were performed with circAXIN1 siRNA transfection or co-transfection of circAXIN1 and linear-295aa. **k** Linear AXIN1-295aa elevates the downregulation of genes downstream of the Wnt/β-catenin pathway caused by circAXIN1 siRNA transfection

Given that AXIN1-295aa competitively bound to APC, we speculated that AXIN1-295aa activates the canonical Wnt/β-catenin signaling pathway. Using a TOPFlash assay, circAXIN1 was found to induce Wnt/β-catenin signaling activation in both 293 T and AGS cells (Fig. 8a). Similarly, linearly expressed AXIN1-295aa was also found to activate Wnt activity (Fig. 8b), whereas circAXIN1 siRNAs decreased the TCF-dependent transcription (Fig. 8c), indicating that circAXIN1 and AXIN1-295aa activated the Wnt/β-catenin signaling pathway. To further confirm this speculation, we identified the nuclear localization of β-catenin and found that circAXIN1 siRNAs caused a reduction in cytoplasmic and nuclear localized β-catenin (Fig. 8d). In agreement with the immunoblotting results, the confocal immunostaining results for β-catenin showed that more β-catenin translocated to the nucleus when there were elevated levels of circAXIN1 and a Wnt agonist (Fig. 8e). We detected a reduced level of β-catenin in the nuclei of cells transfected with circAXIN1 siRNA or Wnt inhibitor XAV939 (Fig. 8e). Furthermore, the overexpression of circAXIN1 induced the expression of Wnt signaling-dependent genes, including Met, c-Myc, LEF1, MMP7, TCF1, CD44, and c-Jun. Some genes, like cyclinD1, remained intact, which suggests that circAXIN1 induces a particular set of Wnt-dependent gene expression (Fig. 8f). Conversely, circAXIN1 siRNAs were found to cause a downregulation of Wnt-dependent genes at the protein and mRNA levels, including c-Myc, c-Jun, CD44, Met, and cyclinD1 (Fig. 8g and h, respectively). We chose c-Jun, c-Myc, and CD44 to investigate whether the expression was truly regulated by the binding of β-catenin to its promoter. The ChIP results revealed that the overexpression of circAXIN1 led to the enhanced binding of β-catenin to these gene promoters (Fig. 8i). To confirm that circAXIN1 activates the Wnt pathway by expressing AXIN1-295aa, we expressed linear AXIN1-295aa together with circAXIN1 siRNA transfection. The β-catenin-dependent transcription was found to be repressed by circAXIN1 siRNA, and the additional expression of linear AXIN1-295aa was found to restore the inhibitory effect (Fig. 8j). Similarly, Wnt-dependent genes were downregulated by circAXIN1 siRNA transfection and upregulated by the supplemented expression of AXIN1-295aa, which indicated that circAXIN1 activates the Wnt/β-catenin signaling pathway by encoding AXIN1-295aa (Fig. 8k).

### CircAXIN1 is oncogenic in vivo

**Fig. 9:**
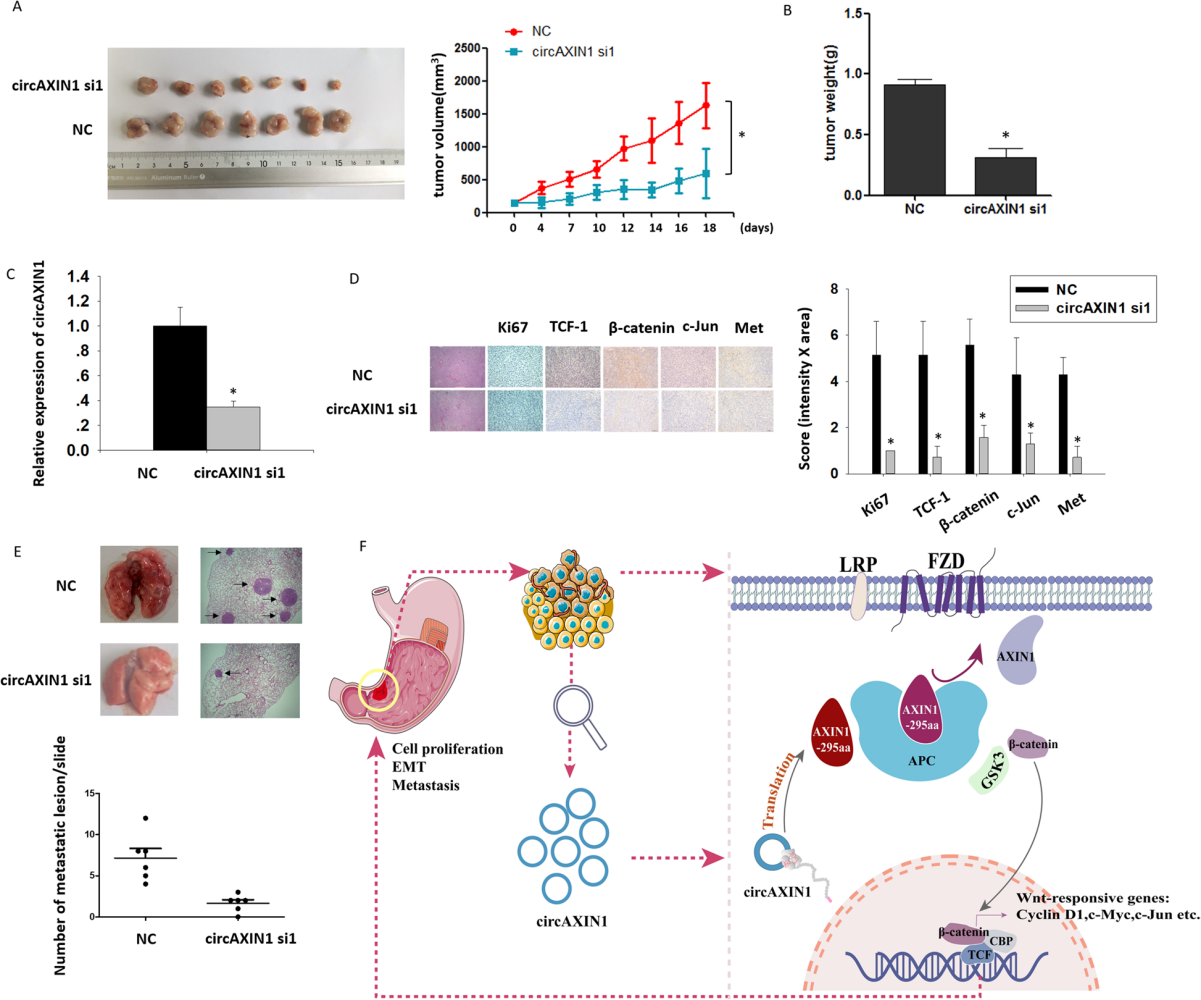
Inhibition of circAXIN1 suppresses tumor growth and metastasis of GC in vivo. **a** Injection of cholesterol-conjugated circAXIN1 siRNA to the subcutaneously formed tumors significantly reduces tumor size. Tumors were treated with negative control (NC) or circAXIN1 siRNA1 and tumor volume was measured and calculated every 2 to 3 days. **b** Tumor weights of the NC and circAXIN1-siRNA1 groups (*n* = 7). **c** The expression of circAXIN1 is reduced in resected tumors from the circAXIN1-siRNA1 treated group. **d** The expression of Ki67,TCF-1, β-catenin, c-Jun, and Met is significantly reduced in circAXIN1-siRNA1-treated tumors. Representative images of immunohistochemistry assays performed on tumor sections from the NC and circAXIN1 siRNA1 groups. **e** Tail vein injection of circAXIN1 siRNA1 inhibits the lung metastasis of GC cells in mice. Lung metastasis lesions were stained with H&E and the number of metastatic lesions was counted. **f** A graphical abstract of the findings. CircAXIN1 is highly expressed and is associated with lymph node metastasis in GC. CircAXIN1 encodes a functional peptide, AXIN1-295aa, which competitively binds to APC, leading to the release and nuclear translocation of β-catenin. Eventually, β-catenin transactivates the canonical Wnt pathway and induces the expression of Wnt-dependent genes to promote cell proliferation and migration

To investigate the oncogenic role of circAXIN1 in vivo, we subcutaneously injected mice with AGS cells to create a xenograft. Cholesterol-conjugated siRNA specifically targeting circAXIN1 was injected into an area surrounding the tumor twice a week for 3 weeks. As shown in Fig. 9a and b, the respective tumor volumes and weights in mice treated with circAXIN1 siRNA were significantly reduced compared with those in the control group. The expression of Wnt downstream genes in the xenograft tumor was observed. The circAXIN1 level was found to be successfully knocked down by the siRNA injections (Fig. 9c). Quantitative immunohistochemistry (IHC) tests showed that the expression levels of Ki67, TCF-1, β-catenin, c-Jun, and Met were inhibited in circAXIN1 siRNA-treated tumors (Fig. 9d). In addition, the administration of siRNA significantly decreased the metastatic colonies in the lungs of nude mice injected with AGS cells through the tail vein (Fig. 9e). These results showed that the siRNA-mediated knockdown of circAXIN1 inhibited GC tumorigenesis and metastasis, which suggests that circAXIN1 is oncogenic in vivo.

In summary, our results indicated that circAXIN1 is highly expressed in GC tissues and that circAXIN1 encodes a novel protein, AXIN1-295aa. The overexpression of circAXIN1 and AXIN1-295aa enhanced the proliferation, migration, and invasion of GC cells, whereas the knockdown of circAXIN1 and AXIN1-295aa inhibited the malignant behaviors of GC cells, in vitro and in vivo. AXIN1-295aa competitively interacted with APC, occupying the AXIN1 position in the destruction complex and consequently activating the Wnt pathway to promote GC development (Fig. 9f).

## Discussion

Although circular RNA was discovered decades ago, it had been overlooked until the recent advances in and application of parallel sequencing. Research has since revealed that circRNAs are conserved, abundant [26], and widespread in eukaryotic cells [27, 28], where they play a vital regulatory role [29]. In addition to serving as microRNA sponges and interacting with protein complexes, circRNAs have been found to possess coding potential [30–32]. The expression level of most circRNAs is not high and only a few contain multiple perfect microRNA binding sites, which suggests that their role as microRNA sponges may not be their main function [5]. Recent studies have revealed that functional peptides can be translated from non-coding RNAs, including primary microRNA (pri-miRNAs), long non-coding RNAs (lncRNAs), and circRNAs, thus blurring the distinction between coding and non-coding RNAs. For example, a 34-amino-acid peptide encoded by the lncRNA Dworf localizes to the sarcoplasmic reticulum membrane and enhances muscle contractility [33]. Regulatory peptides are translated from the plant primaries miR171b and miR165a to delay root development [34]. In addition, evidence has emerged of the existence of functional peptides encoded by circRNAs, including the novel ZNF609 protein isoform from circ-ZNF609 [35], FBXW7-185aa from circ-FBXW7 [36], and β-catenin-370aa from circβ-catenin [37], to name but a few. In the present study, we discovered that circAXIN1 encodes the novel protein AXIN1-295aa to enhance the progression of GC.

Several criteria must be considered when screening functional circRNAs with coding potential. First, circRNA is differentially expressed at a relatively high level in GC, which makes it easily detectable. Second, circRNA must contain a complete ORF, and this ORF should span the junction site. Third, the parental gene is associated with cancer development. Emerging data have revealed that the function of circRNA is highly associated with its parental gene. Fourth, circRNA that is predicted to encode a protein does not necessarily express that protein. Experimental tools must be applied to verify the actual protein expression from circRNAs.

The number of peptides encoded by circRNAs could be underestimated. In our search for potential coding circRNAs, we considered only junction-spanning ORFs, because only they could be distinguished from those peptides encoded from their linear counterparts. However, peptides other than junction-spanning ORFs might also be translated from circRNAs. Ribosomal profiling, recognized to be the most reliable method for identifying coding circRNAs, has been used to identify translatable circRNAs. We also used ribosomal sequencing to search for coding circRNA; however, we found that the junction site of circAXIN1 was not bound to a ribosome, probably due to the excessively stringent conditions of the specific junction site protected by the ribosome in the ribosome profiling sequencing (data not published). Therefore, although the ribosome profile is the most trusted means for identifying novel coding circRNAs, some translatable circRNAs could be missed.

The functions of these novel proteins remain largely unknown. Most proteins translated from circRNAs share their N-terminus with proteins encoded by their parental genes, which generates decoys or competitors for the parental proteins. For example, the peptide AKT3-174aa shares an N-terminus with AKT3 and functions as a decoy for AKT3, thus playing a negative regulatory role in modulating PI3K/AKT signaling activity [10]. The translation of circRNA increases upon stress stimulation [31]. We speculate that circRNA translation may be enhanced during cancer development, taking GC as an example, from early long-term inflammation, intestinal metaplasia, and carcinoma in situ, to distant metastasis. This hypothesis deserves further investigation.

By directly binding to all of the other core components, i.e., β-catenin, APC, CKα, and GSK3β, AXIN1 acts as the central scaffold of the Wnt-pathway destruction complex [14, 38]. AXIN1 is presumed to be a tumor suppressor [39, 40] in many types of cancers, especially colorectal cancer (CRC) [41, 42]. Both somatic and germline mutations in the AXIN1/2 genes have been found in a subset of CRCs and in several other cancer types. AXIN1, because it is the least abundant component, plays a rate-limiting role in the regulation of Wnt activity [43]. AXIN1-295aa contains the APC binding site RGS domain only; it does not contain the β-catenin and GSK3β binding domains or the oligomerization domain. However, from the MS and co-IP results, we determined that AXIN1-295aa also interacts with β-catenin and GSK3β. We propose that AXIN1-295aa binds indirectly to β-catenin and GSK3β by binding to APC. We assume that AXIN1-295aa saturates the available APC, leaving AXIN1, CKα, and GSK3β unable to form the normal destruction complex. β-catenin subsequently translocates to the nucleus and activates downstream genes.

The high stability of circRNA makes it a good candidate as a biomarker [44]. In addition, we found that the expression of circAXIN1 was significantly higher in GC tissues than normal tissues and was associated with tumor invasion depth, differentiation, tumor stage, and lymph node metastasis, which implies that circAXIN1 has the potential to serve as a biomarker for GC prognosis. However, this proposal is formulated based on a limited sample size. Further research must be conducted to confirm that circAXIN1 can be used as a prognostic factor. Furthermore, circAXIN1 siRNA exhibited an excellent therapeutic effect in xenografts and a lung metastasis model, with no severe adverse effects. This result has inspired us to search for the fundamental molecular mechanism accounting for this therapeutic effect. Although in this study we only tested circAXIN1 siRNA in mice, we believe that in the future there will be increasing circRNA siRNA-based therapies in clinical trials and subsequent use [45].

This study is the first to determine that circAXIN1 is translatable and promotes tumorigenesis and aggressiveness in GC. We found circAXIN1 to be highly expressed and associated with lymph node metastasis in GC. We showed that circAXIN1 encodes the functional peptide AXIN1-295aa, which competitively binds to APC, leading to the release and nuclear translocation of β-catenin. Ultimately, β-catenin transactivates the canonical Wnt pathway and induces the expression of Wnt-dependent genes to promote cell proliferation and migration.

## Supplementary Information

**Additional file 1: Supplementary Fig. 1.** Transcript Per Million (TPM) value of circAXIN1(a) and linear AXIN1(b) from five paired GC samples. **Supplementary Fig. 2.** Identification of AXIN1-295aa in 293T with circAXIN1 transfection. **Supplementary Fig. 3.** circAXIN1 and AXIN1-295aa promote cell migration in N87.

**Additional file 2: Supplementary Table 1.** Primer sequences for PCR.

**Additional file 3: Supplementary Table 2.** Upregulated circRNAs in at least three out of five samples.

## Data Availability

Other data that support the findings of this study are available from the corresponding author on reasonable request.
