# Supplementary material for: A novel protein AXIN1-295aa encoded by circAXIN1 activates the Wnt/β-catenin signaling pathway to promote gastric cancer progression
Source: Mol Cancer. 2021 Dec 4;20:158. doi: 10.1186/s12943-021-01457-w (PMC8642992; doi:10.1186/s12943-021-01457-w)
Supplement: Supplementary file 1 — Additional file 1: Supplementary Fig. 1. Transcript Per Million (TPM) value of circAXIN1(a) and linear AXIN1(b) from five paired GC samples. Supplementary Fig. 2. Identification of AXIN1-295aa in 293T with circAXIN1 transfection. Supplementary Fig. 3. circAXIN1 and AXIN1-295aa promote cell migration in N87. [file 12943_2021_1457_MOESM1_ESM.pptx]

## Slide 1
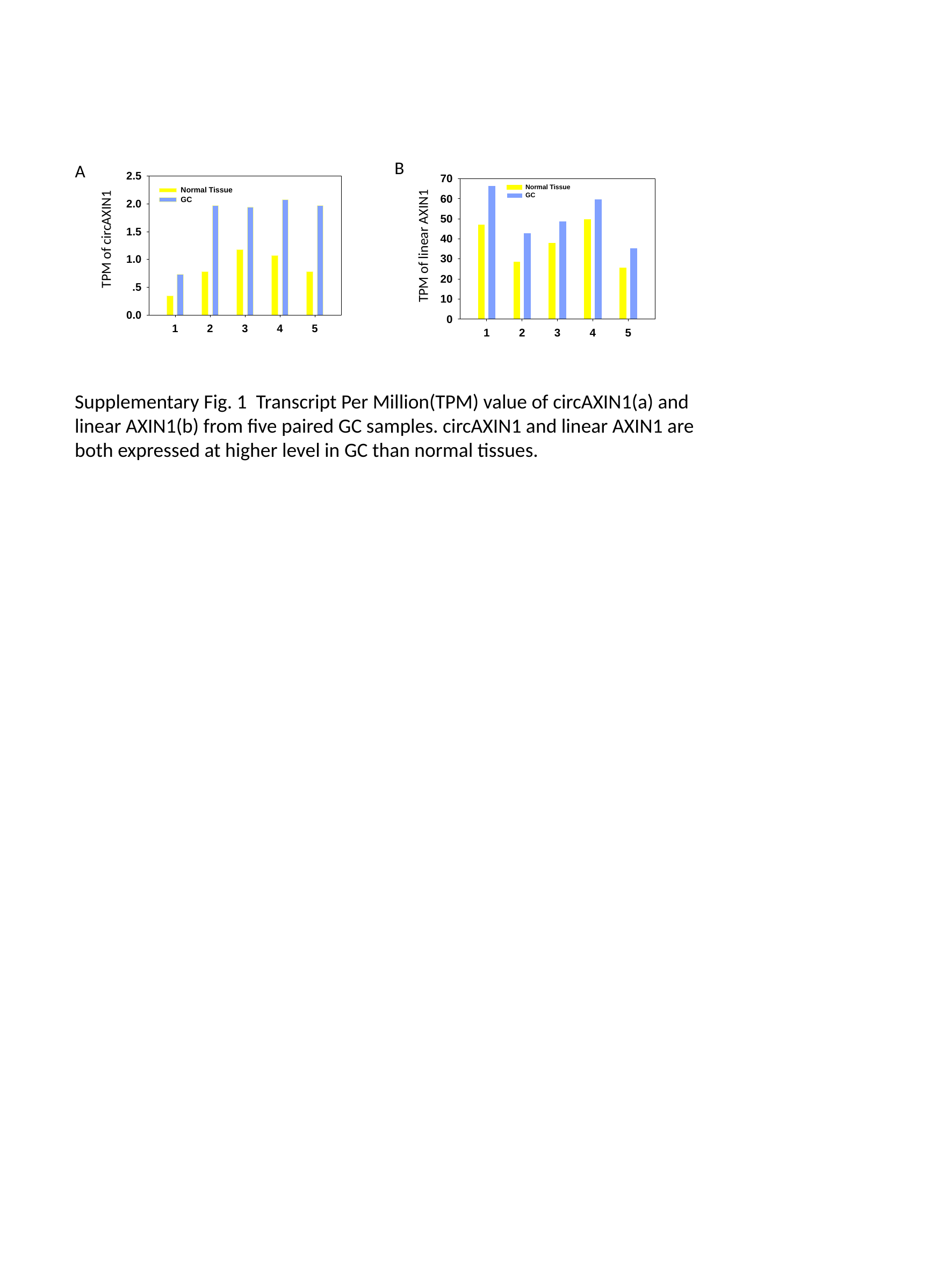

B
A
TPM of circAXIN1
TPM of linear AXIN1
Supplementary Fig. 1 Transcript Per Million(TPM) value of circAXIN1(a) and linear AXIN1(b) from five paired GC samples. circAXIN1 and linear AXIN1 are both expressed at higher level in GC than normal tissues.

## Slide 2
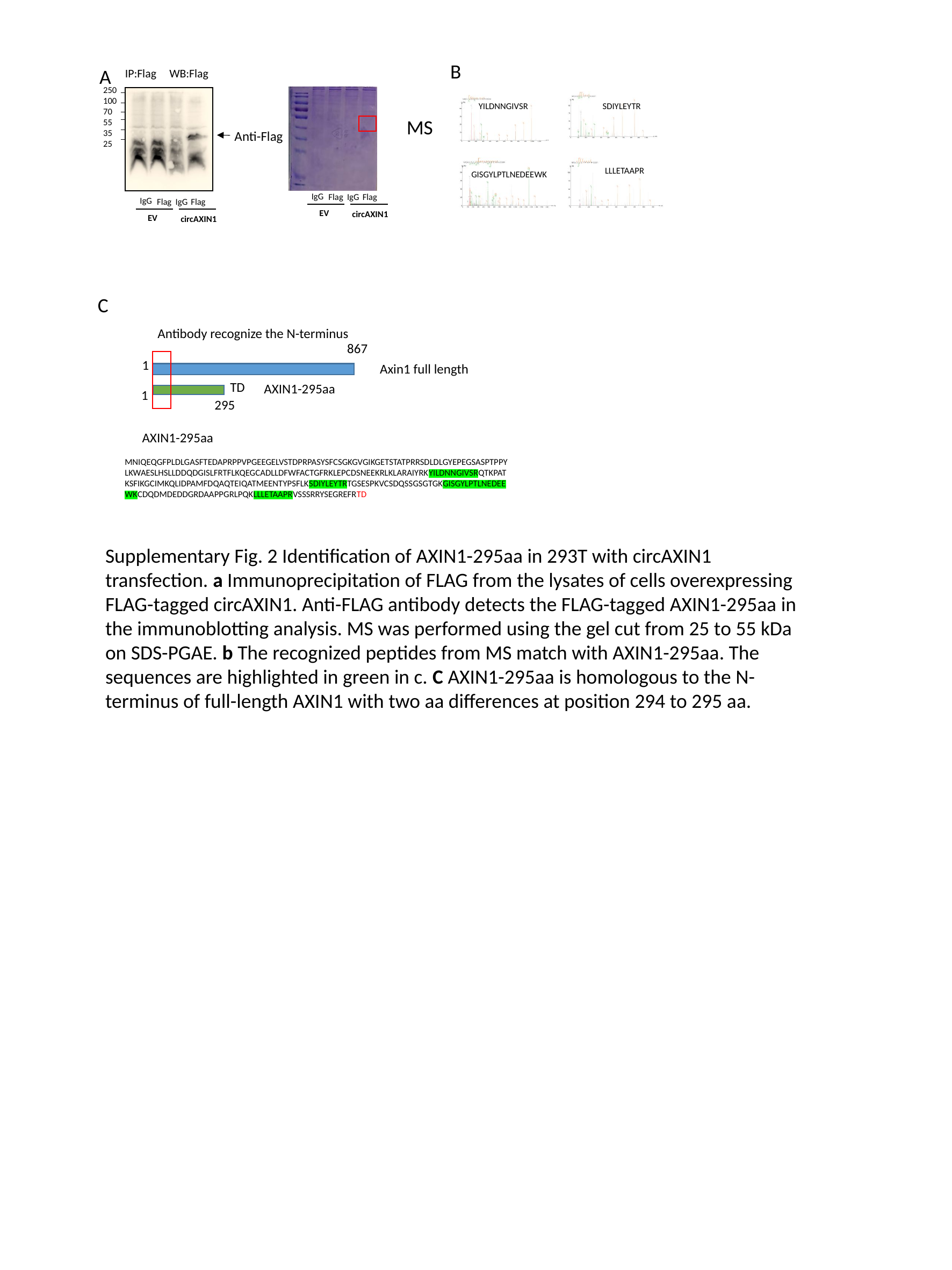

B
A
WB:Flag
IP:Flag
250
100
70
55
35
25
IgG
Flag
Flag
IgG
EV
circAXIN1
YILDNNGIVSR
SDIYLEYTR
MS
Anti-Flag
LLLETAAPR
GISGYLPTLNEDEEWK
IgG
Flag
Flag
IgG
EV
circAXIN1
C
Antibody recognize the N-terminus
1
Axin1 full length
TD
AXIN1-295aa
295
AXIN1-295aa
MNIQEQGFPLDLGASFTEDAPRPPVPGEEGELVSTDPRPASYSFCSGKGVGIKGETSTATPRRSDLDLGYEPEGSASPTPPYLKWAESLHSLLDDQDGISLFRTFLKQEGCADLLDFWFACTGFRKLEPCDSNEEKRLKLARAIYRKYILDNNGIVSRQTKPATKSFIKGCIMKQLIDPAMFDQAQTEIQATMEENTYPSFLKSDIYLEYTRTGSESPKVCSDQSSGSGTGKGISGYLPTLNEDEEWKCDQDMDEDDGRDAAPPGRLPQKLLLETAAPRVSSSRRYSEGREFRTD
867
1
Supplementary Fig. 2 Identification of AXIN1-295aa in 293T with circAXIN1 transfection. a Immunoprecipitation of FLAG from the lysates of cells overexpressing FLAG-tagged circAXIN1. Anti-FLAG antibody detects the FLAG-tagged AXIN1-295aa in the immunoblotting analysis. MS was performed using the gel cut from 25 to 55 kDa on SDS-PGAE. b The recognized peptides from MS match with AXIN1-295aa. The sequences are highlighted in green in c. C AXIN1-295aa is homologous to the N-terminus of full-length AXIN1 with two aa differences at position 294 to 295 aa.

## Slide 3
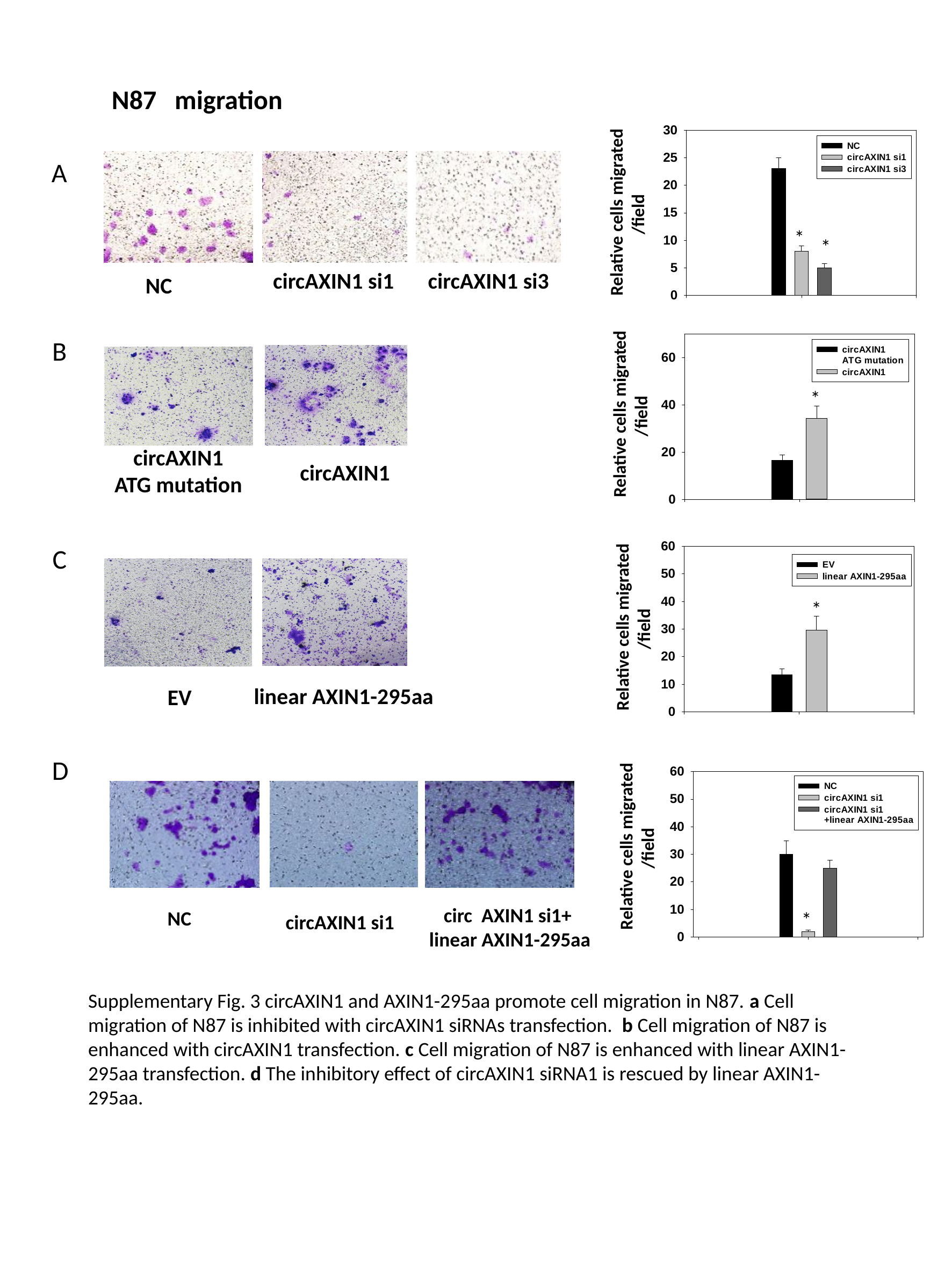

N87 migration
A
 Relative cells migrated
/field
*
*
circAXIN1 si1
circAXIN1 si3
NC
B
*
 Relative cells migrated
/field
circAXIN1
 ATG mutation
circAXIN1
C
*
 Relative cells migrated
/field
linear AXIN1-295aa
EV
D
 Relative cells migrated
/field
circ AXIN1 si1+
linear AXIN1-295aa
NC
circAXIN1 si1
*
Supplementary Fig. 3 circAXIN1 and AXIN1-295aa promote cell migration in N87. a Cell migration of N87 is inhibited with circAXIN1 siRNAs transfection. b Cell migration of N87 is enhanced with circAXIN1 transfection. c Cell migration of N87 is enhanced with linear AXIN1-295aa transfection. d The inhibitory effect of circAXIN1 siRNA1 is rescued by linear AXIN1-295aa.
